# Effect of natural polymer materials on skin healing based on internal wound microenvironment: a review

**DOI:** 10.3389/fchem.2023.1257915

**Published:** 2023-09-05

**Authors:** Ying Yang, Bingbing Li, Mengxin Wang, Shicong Pan, Yu Wang, Jinhui Gu

**Affiliations:** ^1^ The People’s Hospital of SND, Suzhou, Jiangsu, China; ^2^ Nanjing University of Chinese Medicine, Nanjing, Jiangsu, China; ^3^ Guzhou University of Traditional Chinese Medicine, Guiyang, Guizhou, China; ^4^ Suzhou Hospital of Traditional Chinese Medicine Affiliated to Nanjing University of Chinese Medicine, Suzhou, Jiangsu, China

**Keywords:** natural polymer materials, wound internal environment, collagen, hyaluronic acid, chitosan

## Abstract

The concept of wound microenvironment has been discussed for a long time. However, the mechanism of the internal microenvironment is relatively little studied. Here, we present a systematic discussion on the mechanism of natural polymer materials such as chitosan, cellulose, collagen and hyaluronic acid through their effects on the internal wound microenvironment and regulation of wound healing, in order to more comprehensively explain the concept of wound microenvironment and provide a reference for further innovative clinical for the preparation and application of wound healing agents.

## 1 Introduction to wound healing

The wound microenvironment is often divided into external and internal microenvironment (Kruse et al., 2015). The external microenvironment is immediately adjacent to the wound surface, and the cells and extracellular matrix adjacent to it below the wound surface are referred to as the internal microenvironment. The external microenvironment, referred to as the external environment, includes temperature, pH, oxygen, etc., which indirectly influence the internal microenvironment. The internal microenvironment, referred to as the internal environment, includes cells and the extracellular matrix (ECM) containing cytokines, which play a key role in wound healing. The internal and external environments of the wound are dynamic and interact in the wound healing process. The influence of the external wound environment on wound healing has been discussed and studied, but this paper focuses on the influence of natural polymer materials on wound healing based on the internal environment.

**FIGURE 1:**
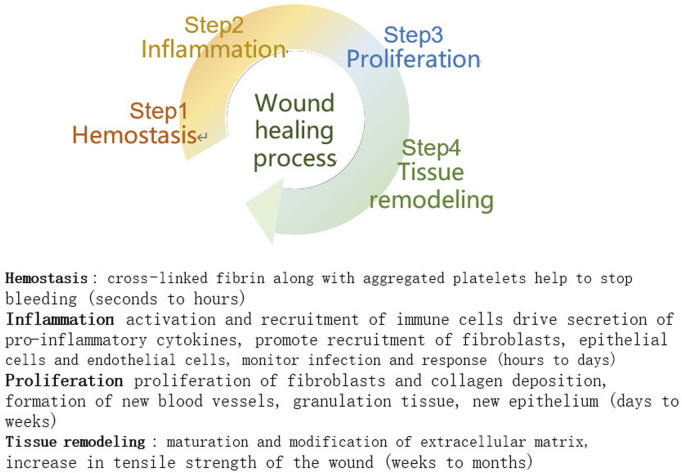
Stages of wound healing (Mathew-Steiner et al., 2021).

Wound healing requires four phases in sequential phases: hemostasis, inflammation, proliferation, and tissue remodelling (Zhao and Fang, 2012), and the phases may overlap in time (Figure 1). In the hemostatic phase, platelets aggregate and the coagulation cascade is activated. A fibrin clot is formed, which acts as a barrier to external factors and prevents further infection while providing a scaffold for cell attachment and proliferation. During the inflammatory phase, inflammatory cells kill microorganisms for early wound debridement and cytokines promote cell proliferation at the site of injury. During the cell proliferation phase, fibroblasts proliferate to synthesize granulation tissue and extracellular matrix, endothelial cells stimulate angiogenesis to restore normal blood circulation to the damaged area, and keratin-forming cells are the main cellular components of the outermost barrier and are used to restore the barrier function. Once the wound is closed, it can enter the final stage of tissue remodelling, mainly collagen deposition, remodelling, and collagen fibre production, and the wound will gradually contract and regain a certain tensile strength to maintain the integrity of the skin surface (Broughton et al., 2006; Xue and Jackson, 2015).

In addition, cytokines as well as enzymes are required at all stages of healing to facilitate communication between cells and the extracellular matrix, which in turn promotes wound healing. Components of the extracellular matrix play an important role in wound healing by assisting integrins in regulating cell signalling, adhesion, proliferation, and migration (Sharma et al., 2021).

## 2 The effects of natural polymer materials on the internal wound microenvironment

Natural macromolecular compounds are large organic compounds that occur naturally in natural organisms. Natural biological macromolecules have low antigenicity and often have specific recognition sites for cell surface receptors that are less likely to cause immune rejection by the body, while some natural polymeric materials can induce and regulate cell growth and differentiation, and have significant effects on their internal microenvironment during the wound healing process. Natural polymer materials are composed of natural polymer compounds as a matrix with other additives (auxiliaries) (Gao and Li, 2002), and have better biosafety, degradability and physicochemical properties than synthetic materials (Li et al., 2016). In addition, natural polymeric materials are now widely used in soft tissues treatment due to their wide availability, low cost, and economic advantages (Wang et al., 2009). The main ones currently used for skin tissue repair and wound healing are polysaccharides and proteins. Polysaccharides include starch, chitosan, cellulose, hyaluronic acid, and proteins include collagen. The author searched PubMed and CNKI databases using the keywords “natural polymers, skin, wound healing” and “natural polymer materials, skin, wound healing.” Relevant literature published between 2018 and 2022 was searched. A total of 508 relevant papers were retrieved, including 2 in Chinese and 506 in English. After excluding 336 papers with low relevance to the study and other old and repetitive content, the remaining 172 papers were found to have a high frequency of chitosan, cellulose, hyaluronic acid and collagen, which are currently the main natural materials used in the field of skin repair. Chitosan is a natural alkaline polysaccharide that can be extracted from bacteria or crustaceans (Lu et al., 2020). Cellulose is a natural linear polysaccharide, one of the most abundant natural polymers produced by plants or bacteria (Lynd et al., 2002). Collagen is the most abundant animal protein, widely distributed in connective tissues, accounting for approximately 1/3 of human proteins and 3/4 of the dry weight of skin (Chattopadhyay and Raines, 2014). Hyaluronic acid (HA) is a naturally occurring linear polysaccharide and a major component of the extracellular matrix. Wound dressings such as hydrogels, sponges, films and tissue scaffolds made from them are now widely used in the field of wound repair and regeneration. In this review, we take chitosan, cellulose, collagen and hyaluronic acid (HA) as examples to elucidate their effects on the internal microenvironment of wounds.

### 2.1 Promotion of platelet aggregation and fibrous clot formation

Natural polymeric materials can affect the internal microenvironment of the wound by promoting platelet aggregation and fibrous clot formation. Chitosan, a positively charged natural polysaccharide, can interact with a variety of negatively charged substances on the surface of activated platelets to promote platelet aggregation (Jackson et al., 2005), and with negatively charged groups on the surface of red blood cells to promote fibrin clot formation and red blood cell aggregation (Ong et al., 2008). Chitosan enhances the expression of platelet membrane glycoprotein GPIIb/IIIa receptors, and the activated GPIIb/IIIa binds to collagen and vascular hemophilia factor (VWF) to anchor platelets to the collagen surface and form platelet plugs, thereby promoting platelet adhesion and aggregation (Jackson et al., 2005; Lord et al., 2011) and achieving hemostasis. In addition, chitosan also prevents the rapid degradation of fibrin clots by inhibiting the activation of fibrinolytic enzymes, thereby achieving hemostasis.

Oxidized cellulose is a polymer naturally derived from cellulose, and oxidized cellulose-based hemostatic materials have been shown to have good hemostatic efficacy, but the core of the mechanism of action remains unclear. Existing studies have shown that on the one hand, oxidized cellulose may act on active bleeding sites to trap active components in the blood and increase the concentration of clotting factors, thereby accelerating the clotting process. On the other hand, the carboxyl group of oxidized cellulose lowers the pH, which leads to non-specific aggregation of platelets and thus the formation of artificial clots. In addition, carboxyl groups can produce acid mediators that convert hemoglobin to acid hemoglobin, releasing Fe3^+^ which binds to the carboxyl groups and accelerates clot formation, blocking blood flow (Zhang et al., 2020).

The hemostatic effect of collagen was discovered as early as 1953. Exposed collagen after injury can cause platelet aggregation, secrete factors that stimulate the coagulation cascade reaction, lead to fibrin clot formation and produce stable hemostatic clots, thus stopping the initial bleeding (Xue and Jackson, 2015). Hemostatic materials were made from collagen in 1982 and were shown to have good hemostatic properties (Zhang, 2015). In a study using resorbable collagen for hemostasis in general surgery patients, the mean time to hemostasis was 2.2 min, with significant hemostatic effects in terms of massive capillary, venous, and small arterial bleeding without adverse effects such as foreign body reaction, infection, or allergy (Browder and Litwin, 1986).

During the hemostatic phase, platelets secrete large amounts of high molecular weight hyaluronic acid (HMW-HA). HMW-HA is a structural substrate for fibrin deposition, and HMW-HA binds to fibrinogen to promote fibrinogen deposition and clot formation to achieve hemostasis (Yang et al., 2021). Zheng et al. (2022) used resorbable hyaluronic acid hemostatic material in a canine uterine bleeding modeland observed a significant reduction in bleeding time and volume of trauma, with good hemostatic effect.

### 2.2 Recruitment of inflammatory cells and release of inflammatory factors

Inflammation is an important step in the wound healing process, promoting cell synthesis and proliferation, while the timely resolution of inflammation is equally important to facilitate the transition to the proliferation and remodeling phase of the wound. Natural polymers such as chitosan can promote the recruitment of inflammatory cells and the release of inflammatory factors during the inflammatory phase, and enhance the inflammatory phase response. Chitosan has immunomodulatory effects and can induce and regulate immune cells by altering the microenvironment of the immune system, thereby achieving therapeutic effects through modulating immune function in the skin and soft tissues. In addition, related studies have shown that chitosan can induce cytokine secretion from NK cells, synthesis and secretion of interleukin (IL)-8, and accelerate neutrophil exudation (Ni and Hu, 2012). In a study by Chen et al. (2021), hydrogels containing chitosan could promote skin wound healing in diabetic rats by downregulating proinflammatory factors such as TNF-α and interleukin (IL)-1β.

Unlike chitosan, cellulose has no intrinsic antimicrobial activity, and the antimicrobial properties are mostly enhanced by surface modification with a variety of functional groups, such as carboxyl groups, aldehydes, amines, alkylamines and quaternary ammonium groups. Or this property is enhanced by chemical modification of organic bioactive molecules, such as antibiotics, antimicrobial peptides, phage, etc., (Abdelhamid and Mathew, 2022).

Type I and IV collagen can act as neutrophil elicitors (Ricard-Blum and Ballut, 2021), promoting neutrophil recruitment, enhancing phagocytosis and immune response, and thus participating in the inflammatory response. Soluble fragments from collagen degradation can also recruit immune cells such as macrophages to remove inactivated tissue and facilitate the transition from the inflammatory to the proliferative phase. In one study, collagen matrix dressings were applied to mouse wounds and a strong inflammatory response with the ability to maximize wound inflammation, while the regression was robust and short-lived, preparing the wound for re-epithelialization and early healing (El et al., 2019).

HA as a major component of edema fluid promotes neutrophil recruitment, participates in phagocytic debris and necrotic tissue removal, and releases inflammatory factors such as TNF-α, IL-1β, and IL-8 (Tavianatou et al., 2019), enhancing the inflammatory phase of the response. The secretion of these inflammatory factors also promotes the conversion of HMW-HA to low-molecular-weight hyaluronic acid (LWM-HA), which in turn binds to CD44 receptors on the surface of monocytes and granulocytes (Wolny et al., 2010), participating in the recruitment of leukocytes and monocytes and enhancing the local inflammatory response. LWM-HA also interacts with toll-like receptors on lymphocytes and macrophages (Aya and Stern, 2014), which in turn promotes the expression of inflammatory factors.

### 2.3 Regulation of enzyme activation

Degradation of the ECM is an important part of wound healing and scar formation. Matrix metalloproteinases (MMPs) catalyze the hydrolysis of key ECM molecules, including collagen, elastin, laminin, and fibronectin. In addition to matrix degradation, MMPs influence many biological functions by regulating growth factors and their receptors, cytokines and chemokines, and other enzymes (Xue and Jackson, 2015). An imbalance in the level of activation of these enzymes leads to increased destruction of ECM components and inappropriate activation of soluble mediators, resulting in chronic wounds that do not heal easily (Mathew-Steiner et al., 2021). Natural polymers can promote wound healing by regulating the level of enzyme activation.

Chitosan inhibits the secretion of fibrinogen activators, thereby increasing the level of fibrin inhibition. It has been reported (Fukasawa et al., 1992) that 80% deacetylated chitosan membranes can inhibit the level of fibrinogen activation by inhibiting the secretion of fibrinogen activator by macrophages, prolonging the presence of fibrin clots and thus achieving hemostasis (Feng et al., 2021).

In MMP, collagenase and gelase degrade intact and damaged protofibrillar collagen, respectively, and are essential for collagen renewal during wound healing (Mathew-Steiner et al., 2021). Collagen I and III are preferentially cleaved by MMP-1 (also known as collagenase-1) and MMP-8 (collagenase-2), whereas collagen IV is degraded by the gelatinase MMP-9. In addition these enzymes contribute to the release of bioactive fragments from collagen, such as endothelial inhibitors (Ricard-Blum and Ballut, 2021). Collagen can both act as an inducer of enzymes, down-regulating the level of enzyme activation and also rebuild tissue structure during the healing process through the bioactive fragments released by the enzymes (Mathew-Steiner et al., 2021).

Fibrinolytic enzymes are involved in the proteolytic cascade reaction of MMP and other proteases that cause tissue damage during inflammation (Trabucchi et al., 2002). The inflammatory factor TNF-α expressed by HA during the inflammatory phase can stimulate the production of the HA-binding protein TSG-6, which forms a stable complex TSG-6-IαI-HA with inter-α trypsin inhibitor (IαI) (Wisniewski et al., 2005). In this way, HA promotes fibrinolysis inhibition, which in turn limits the inflammatory response and allows its timely regression, paving the way for subsequent wound healing.

Proteases in the wound microenvironment inhibit the degradation of growth factors and ECM in chronic wounds and reduce their efficacy for wound healing, while cellulose and its derivatives can be used as protease inhibitor carriers with their neutralization. Nanocellulose-based protease sensors and protease-regulating dressings can be used for the immediate detection of proteases (Frazier et al., 2020), increasing the efficacy of wound care and improving the healing of chronic wounds.

### 2.4 Involved in free radical regulation

The generation of free radicals produced locally after skin injury can act as secondary messengers for immune cells and non-lymphocytes to promote tissue repair and prevent bacterial colonization. However, excessive free radical release can damage healthy epithelial cells and interfere with wound healing (Baron et al., 2020). Some natural polymers can be involved in the regulation of free radicals to prevent bacterial contamination, but also the risk of damage to the epithelial cells, its specific regulatory mechanism and regulatory range still need more in-depth study.

The amino and hydroxyl groups in the molecular chain of chitosan can effectively scavenge excess free radicals in the body (Verlee et al., 2017). The effect of chitosan is related to the relative molecular weight and acetylation level, and chitosan shows greater ability in scavenging free radicals with relatively low molecular weight and high acetylation level.

Collagen showed scavenging effects on DPPH radicals and OH radicals, and showed a quantitative-effect relationship within a certain mass concentration range. Collagen extracted from monkfish skin, squid skin, deer skin and fish scales showed free radical scavenging effects *in vivo* and *in vitro* (Zhu et al., 2012; Hu et al., 2014; Ma and Yang, 2015; Song et al., 2018). Observing hydrolysates of collagen from sea cucumber hydrolysates, Abedin et al. (2014) suggested that the peptides may be reacting with free radicals to terminate the chain reaction and produce a more stable product that exhibits good antioxidant properties, which helps to improve the internal environment of wounds, thus promoting wound healing.

As a natural compound, HA can be oxidized and degraded by free radicals to hyaluronic acid, which effectively fights against oxygen free radicals through its own rapid metabolism, achieving the purpose of scavenging free radicals in the body (Chen, 2007), thus protecting the granulation tissue from oxygen free radical damage and stimulating wound healing (Trabucchi et al., 2002). A study in which hyaluronic acid cream and ethyl hyaluronate gel were applied to rat wounds with abnormally elevated oxygen radicals showed that hyaluronic acid and its ethyl ester derivatives significantly improved wound healing and had an antagonistic effect on oxygen radicals.

### 2.5 Extracellular matrix remodeling

The extracellular matrix is the main internal environment for wound healing, and natural polymeric materials mostly have a similar structure to the ECM, or related functional properties, and thus can remodel the extracellular matrix, which is an important part of promoting wound healing. Chitosan has the function of promoting the secretion of cytokines such as TGF-β and PDGF (Feng et al., 2021). Among them, TGF-β can induce macrophages to migrate to the injury site, promote fibroblast proliferation and enhance collagen secretion. PDGF can stimulate fibroblast migration and proliferation, promote the synthesis of ECM components such as glycosaminoglycan, proteoglycan and collagen, and thus achieve the effect of remodeling the ECM (Howling et al., 2001). It was also found that chitosan has the biological activity of selectively promoting epithelial cell growth, stimulating fibroblast proliferation while inhibiting its overproliferation, while the effect on fibroblast proliferation mainly depends on the molecular weight and degree of deacetylation of chitosan (Wang et al., 2009).

The structure of cellulose is similar to that of ECM, and the purest form of cellulose is called bacterial cellulose (BC). It has a unique 3D nanofibrous network structure that facilitates cell penetration and proliferation, particularly promoting the growth of connective tissue cells (Navya et al., 2022). Studies have shown that keratin-forming cells spread and migrate better on the BC surface (Silva et al., 2022).

Collagen, as a structural component of the extracellular matrix, contributes to the stabilization of growth factors and the regulation of cell adhesion, as evidenced by the effective uptake and utilization of fibronectin. Fibronectin-synthesizing protofibrils attach cells to the ECM, and the formed ECM and collagen together form a structural framework that plays an important role in tissue repair (Barnes, 2019). In addition, integrins and discoidin structural domain receptors of collagen can dynamically monitor the extracellular matrix by inducing matrix degradation and renewal, maintaining the integrity of the collagen extracellular matrix and promoting wound extracellular matrix remodelling (Vogel, 2001).

HA is widely distributed in the skin ECM. LMW-HA and fibronectin are regarded as guides of fibroblast migration, and together they direct fibroblast invasion and proliferation and promote fibroblast differentiation into myofibroblasts. Jia et al. (2022) prepared a pH-responsive hyaluronic acid hydrogel for wounds in diabetic mice, and *in vitro* results showed that the hydrogel significantly increased fibroblasts. The *in vitro* results showed that the hydrogel significantly increased the adhesion and infiltration of fibroblasts, effectively promoted the remodeling of ECM, and provided a good internal environment for wound healing, especially for chronic wounds.

### 2.6 Promotion of angiogenesis

Natural polymeric materials can influence the internal microenvironment of a wound by participating in the regeneration of blood vessels. Chitosan can promote the secretion of cytokines such as transforming growth factor β (TGF-β) and platelet-derived growth factor (PDGF) by macrophages (Feng et al., 2021). Among them, TGF-β promotes chemotaxis and proliferation of fibroblasts and endothelial cells during the inflammatory and proliferative phases of wound healing (Liarte and Bernabe-Garcia, 2020). PDGF can recruit pericytes into capillaries, causing capillary constriction, reducing blood pressure and flow rate at the wound site, setting the stage for wound repair and increasing the structural integrity of blood vessels. IL-1 can recruit and activate leukocytes to the wound site, enhancing the inflammatory phase of the response and stimulating angiogenesis.

Hydrolyzed fragments of different collagen types have either a facilitating or inhibiting effect on neovascularization. Among them, type I collagen is effective in stimulating neovascularization *in vitro* and *in vivo* by binding to specific integrin receptors to which the C-prepeptide fragment can recruit endothelial cells (Kisling et al., 2019). The type IV collagen fragment, arresten, binds to α1β1 integrins on endothelial cells and is an inhibitor of angiogenesis in squamous cell carcinoma. In addition, HA can exert anti-angiogenic effects by inhibiting the proteolytic activity of MMP-2 (Kisling et al., 2019; Mathew-Steiner et al., 2021).

HA facilitates the fixation of adipose stem cells and prolongs the residence of their exosomes in the vasculature, and the combination of the two helps to promote vascularization of the trabecular surface (Vasvani et al., 2020). In addition, HMW-HA is able to inhibit early response genes in vascular endothelial cells, which in turn inhibits angiogenesis. In contrast, during wound healing, HMW-HA is rapidly cleaved to the highly angiogenic LMW-HA. LMW-HA can control the expression of vascular endothelial growth factor and participate in the angiogenic response through CD44 and RHAMM receptor-mediated signaling pathways (Aya and Stern, 2014).

### 2.7 Regulation of cell signaling

In addition, some natural polymeric materials can modulate intracellular signaling, which in turn affects the internal microenvironment of the wound by influencing cellular behavior. Collagen is a receptor-mediated signaling molecule that independently triggers various signaling pathways through three types of surface receptors, integrins, discoidal protein structural domain receptors and glycoprotein VI, when bound to induce cell signaling and define cell shape and behavior, which in turn promotes platelet aggregation, inflammation regulation, angiogenesis, granulation tissue formation and re-epithelialization. High molecular weight hyaluronic acid has multiple binding sites with CD44 receptors, and their interaction can control intracellular signaling pathways (Litwiniuk et al., 2016), leading to the functions of angiogenesis, cell migration, elimination of intracellular reactive oxygen species and proliferation of extracellular matrix components.

### 2.8 Others

As natural polymeric compounds have different molecular structures and chemical compositions, their physical properties and biological vary, among which chitosan also has good antibacterial effects. Positively charged chitosan can interact with negatively charged phosphopeptides in the cell wall of Gram-positive bacteria and lipopolysaccharide anions on the outer membrane of Gram-negative bacteria to disrupt the bacterial cell wall, causing loss of cell function and eventual death (Abd et al., 2020; Ardean et al., 2021). In addition, chitosan can also cross bacterial cell membranes and interfere with the translation and transcription of bacterial genetic material (Verlee et al., 2017), thus affecting normal cell function and achieving antibacterial effects.

HA has the function of regulating wound re-epithelialization. HA is a component of the basal layer on which keratinizing cells depend, and the presence of LMW-HA at the wound edge interacts with CD44 to promote keratinizing cell proliferation, which in turn regulates the wound re-epithelialization process.

## 3 Clinical applications of natural polymer materials

### 3.1 Chitosan

#### 3.1.1 Chitosan hydrogels

The structure and biological properties of chitosan are similar to those of glycosaminoglycan, the major component of the extracellular matrix (Ye et al., 2002), which has excellent biocompatibility and film-forming properties (Minagawa et al., 2007) and is a good material for trauma repair. Chitosan and its polymers as raw materials have been widely used in the preparation of hydrogels, microneedles, sponges, etc. (Table 1). Hydrogels made from chitosan as raw material have good hydrophilicity, absorption and remarkable antibacterial properties, which help to restore the connection between the dermis and epidermis while also having the effect of promoting the re-epithelialization of whole skin defects (Li et al., 2016), and are widely used clinically as antibacterial dressings. Zhu et al. (2022) applied chitosan hydrogel to infected whole skin defect wounds in diabetic mice, and the chitosan hydrogel was more biosafe and induced a higher level of macrophage polarization than the control hydrogel without chitosan, and the wounds were completely covered by new epidermis with regular cell arrangement, which effectively promoted rapid wound healing. Zhou et al. (2021) used chitosan-polyethylene glycol-tyrosine molecular hydrogel to treat post-open abdominal wounds, and due to its good water retention capacity and moist surface environment (Serafim et al., 2020), it effectively improved the contact microenvironment between the closed abdominal material and the intestinal wall, reduced the intestinal wall abrasion and damage, and significantly reduced the level of oxidative stress in the small intestinal after abdominal opening.

**TABLE 1:** The clinical applications of chitosan.

Raw material	Biomaterial products	Application	References
Chitosan	chitosan hydrogels	antibacterial dressings; total skin defect wounds	Li et al. (2016)
	microneedle array patches	skin wound repair	Serafim et al. (2020)
	sponges	hemostatic materials	Chi et al. (2020)
chitosan-polyethylene glycol-tyrosine molecular	chitosan-polyethylene glycol-tyrosine molecular hydrogel	post-open abdominal wounds	Matica et al. (2019)
Carboxymethyl chitosan-carbon dots (CMCS-CQDAG)	self-healing hydrogel	bacterial infections	Sanad and Abdel-Bar (2017)
chitosan derivatives	self-healing hydrogel	wound	Serafim et al. (2020)
a silk protein-chitosan	3D porous scaffold	muscle embedding	Li et al. (2021)
a composite chitosan	artificial skin	a full skin defect	Zhao et al. (2017)
chitosan and zinc nitrate	a composite microneedle array	skin wound repair	Xie (2015)
chitosan, sodium bicarbonate and collagen	a composite sponge	skin wound hemostatic	Lug et al., 2002

Currently, the main research areas of chitosan hydrogels are self-healing hydrogels, dual network hydrogels and pH-sensitive hydrogels. Among them, self-healing hydrogels can automatically respond to damage, repair their own damage and restore their structure, morphology and properties, and are a hot research topic in the field of biomedical materials (Hou et al., 2022; Qu et al., 2022). Li et al. (2021) used carboxymethyl chitosan carbon dots (CMCS-CQDAG) to prepare self-repairing hydrogels, which can completely self-heal after 3 h at 37°C, their degradation rate. The slow degradation rate facilitates long-term treatment of bacterial infections. Zhao et al. (2017) prepared an injectable conductive self-healing hydrogel based on chitosan derivatives, which had excellent antimicrobial activity, electroactivity, and free radical scavenging ability, and significantly enhanced wound healing by upregulating growth factors involved in wound healing and greatly promoting extracellular matrix synthesis and collagen deposition.

#### 3.1.2 Chitosan skin scaffolds and artificial skin

Scaffold materials and seed cells are key components in tissue engineering research (Langer and Vacanti, 2016), and extracellular matrix and its biomolecules such as collagen can promote vascular and epithelial regeneration by inducing specific cellular behaviors, delivering cells, or binding to specific factors and materials (Luo et al., 2021) Chitosan contains derivable active hydroxyl and amino groups (Che et al., 2008), which can provide many binding sites for cell adhesion and migration, promote communication between cells and extracellular matrix, and enhance repair of damaged sites (Zhang et al., 2011). Meanwhile, chitosan is non-toxic and non-irritant, which is an ideal extracellular matrix material and is now widely used in the field of tissue engineering (Che et al., 2008). Xie Hong (Xie, 2015) prepared a silk protein-chitosan 3D porous scaffold for muscle embedding experiments, and the results showed that the scaffold provided a good environment for cell growth, showed excellent biocompatibility, promoted cell adhesion and growth to the inside of the scaffold, and formed a complete fibrous capsule wall structure around the scaffold, and the synthesized collagen was convergent, which was beneficial for wound healing.

Research on chitosan artificial skin has been carried out since the 1940 s, and the chitosan artificial skin is biocompatible and has hemostatic, analgesic, antibacterial and anti-inflammatory functions. As the wound heals and its own skin grows, the artificial skin can be degraded and absorbed by the body to promote skin regeneration (Wang et al., 2009). Lu et al. (2002) transplanted a composite chitosan artificial skin with dermis and epidermis to a full skin defect in rabbits, and the results showed that the wound healed well without obvious immune rejection, and the epidermal structure was clear and the new fibers and blood vessels increased significantly after 2 months, which was a good experiment.

#### 3.1.3 Chitosan microneedles

Microneedle patches consist of hundreds of micron-sized needles that can deliver drugs directly into the epidermis or dermis without pain. Chitosan microneedles are better suited for topical and transdermal drug delivery due to their good film-forming ability, biodegradability and biocompatibility. In addition, chitosan has a modifiable active amino and hydroxyl functional group, which makes it flexible and tunable and can be tailored to the desired strength and function, thus chitosan has become the material of choice for microneedle fabrication (Gorantla et al., 2021).

Chi et al. (2020) fabricated chitosan-based microneedle array patches that also contained vascular endothelial growth factor. The chitosan microneedle patch had significant antimicrobial properties and faster wound healing effect compared to the control group. Yi et al. (2021) developed a composite microneedle array of chitosan and zinc nitrate, which combined the structural properties of microneedles with the antimicrobial properties of chitosan and zinc nitrate. The microneedle structure increased the contact area between the drug delivery system and the bacterial biofilm by approximately 14%–23%, effectively facilitating drug diffusion. The antimicrobial properties of the composite microneedle were confirmed by inhibiting up to 100% of the bacterial biofilm by CFU counting and bacterial live-dead staining tests. Because the eradication of bacterial biofilm is beneficial for the treatment of chronic wounds, chitosan microneedles can be clinically used for skin wound repair.

#### 3.1.4 Chitosan sponges

Sponges are able to absorb large amounts of wound exudate and maintain a moist environment at the wound site due to their large porosity, good biocompatibility, and contour swelling (Simoes et al., 2018). Chitosan sponges are biodegradable, loaded with antimicrobial drugs and promote blood clotting in wounds (Hu et al., 2018) and are widely used as hemostatic materials (Matica et al., 2019). Also, chitosan sponges are rich in andrographolide, which has a large pore size and expansion rate and can effectively promote wound healing and reduce scar formation (Sanad and Abdel-Bar, 2017). Zhu et al. (2023) prepared a composite sponge containing chitosan, sodium bicarbonate and collagen, which was used in a mouse skin wound model to effectively promote fibroblast proliferation and had a good hemostatic effect when used in a rat tail amputation hemorrhage model. After the sponge was implanted into the back wound of rats for 1 week, there was no obvious inflammatory reaction in the surrounding skin, and the content of new capillaries and collagen was significantly higher than that of the control group, and the wound surface recovered well. The results showed that the chitosan composite sponge has good biocompatibility, antibacterial and hemostatic effects and promotes tissue healing.

### 3.2 Cellulose

#### 3.2.1 Cellulose hydrogel

Currently, the main cellulose used for skin repair is bacterial cellulose (BC), which is produced by various acetic acid bacteria, rhizobia, etc. through oxidative fermentation. It is characterized by high crystallinity, high water-holding capacity, ultrafine nanofiber network, high tensile strength and elastic modulus, and has been widely used in wound healing (Table 2). BC hydrogel on the one hand, meets the requirements of high water content, high porosity, good mechanical properties and biocompatibility of hydrogels, on the other hand, has a high surface area of fiber material, structural advantages of easy weaving and reticulation (Meng et al., 2023). de Amorim et al. (2022) suggested that pure BC hydrogel membranes are very close to human skin and have the function of relieving pain and accelerating the regeneration of epithelial tissues. BC hydrogels can transfer drugs to wounds and act as barriers to protect wounds. Therefore, BC hydrogels have become ideal candidates for wound dressings. BC/chitosan composites were prepared by impregnating BC in chitosan and then lyophilizing the membrane. *In vivo* results showed that BC/chitosan accelerated epithelialization and regeneration of rat skin wounds (Lin et al., 2013).

**TABLE 2:** The clinical applications of cellulose.

Raw material	Biomaterial products	Application	References
bacterial cellulose	BC hydrogels	wound dressings; transfer drugs	de Amorim et al. (2022)
	transdermal patches	drug delivery	Zhao et al. (2021)
	microneedle patches		
bacterial cellulose and chitosan	BC/chitosan hydrogels	wound healing	Lin et al. (2013)
bacterial cellulose and hyaluronic acid	BC/HA membrane	skin injuries	Li et al. (2015)
	BC-supported HA microneedles	micromolding technology	Fonseca et al. (2021)

#### 3.2.2 Cellulose films

BC has a neutral electrostatic charge that facilitates the incorporation of negatively and positively charged bioactive compounds (Chopra et al., 2022), thus promoting their incorporation into the polymer base, making it an ideal wound dressing for biomedical engineering. When used as a membrane, it helps to increase cell adhesion as well as cell proliferation, migration and differentiation, thereby accelerating re-epithelialization and thus the wound healing process (Nicoara et al., 2020). Li et al. (2015) prepared a novel BC/HA membrane that enhanced the proliferation of primary human fibroblasts *in vitro* in Wistar rats and shortened the wound healing time of full-thickness skin injuries.

#### 3.2.3 Cellulose patches

Antimicrobial transdermal patches and microneedle patches are mainly used to deliver antimicrobial and antiviral drugs, bypassing the outermost skin barrier and delivering drugs directly to the epidermis and dermis for sustained drug delivery (Zhao et al., 2021). BC, a biopolymer, has been used as a transdermal patch for synthetic wound dressings due to its biocompatibility, non-toxicity, nano network structure, and good porosity. Experiments have shown that BC-supported hyaluronic acid (HA) microneedles can be used to deliver drugs using micromolding technology without any adverse changes in skin barrier function or stratum corneum hydration (Fonseca et al., 2021).

### 3.3 Collagen

Collagen has good surface activity and can be prepared as sponges, hydrogels or films, which is an important material for new dressings (Table 3).

**TABLE 3:** The clinical applications of collagen.

Raw material	Biomaterial products	Application	References
Collagen	collagen sponge	surgical hemostasis	Zhu (2021)
	Collagen films	wound barriers	Felgueiras and Amorim (2017)
	scaffolds for antibiotics and exogenous growth factors	*ex vivo* tissue engineering and direct implants	Chattopadhyay and Raines (2014)
	collagen sponge artificial dermis	soft tissue defects in the skin after squamous cell carcinoma surgery	Hao et al.,. 2013
protofibrillar collagen and gelatin	modified sponge	an artificial skin graft	Koide et al. (1993)
bovine type I collagen	collagen sponge scaffold	the regeneration of skin wound tissue	Jin et al. (2008)
tilapia fish skin	collagen hydrogels	deep second-degree scald wounds	Wang et al. (2019a)
recombinant human type III collagen	recombinant human type III collagen hydrogel	total skin defect wounds	Liu et al. (2019)
porcine collagen	collagen films	burn wounds	Gao et al. (1992)
silver sulfadiazine collagen	compound silver sulfadiazine collagen burn film	deep burn wounds	La et al. (2001)

#### 3.3.1 Collagen sponge

Collagen sponge has a high moisture content and can absorb a large amount of exudate and adhere smoothly to the wound bed. Meanwhile, collagen can promote platelet aggregation and activate the coagulation cascade reaction, so collagen sponges have good hemostatic properties and are now widely used for surgical hemostasis (Zhu, 2021). In addition, collagen sponges can also be used as scaffolds for antibiotics and exogenous growth factors (Chattopadhyay and Raines, 2014) for *in vitro* cell culture, mainly for *ex vivo* tissue engineering and direct implantation, and a modified sponge that can be used as an artificial skin graft was developed in 1993 by combining protofibrillar collagen with gelatin (Koide et al., 1993). Jin et al. (2008) prepared a collagen sponge scaffold from bovine type I collagen with a porous mesh structure in its cut surface, which can be used as a support and attachment point for epidermal cell growth and has the condition to guide the regeneration of skin wound tissue. Hao et al. (2013) used collagen sponge artificial dermis to repair soft tissue defects in the skin after squamous cell carcinoma surgery, and the results showed rapid proliferation of granulation tissue and good wound healing.

#### 3.3.2 Collagen hydrogels

Collagen hydrogels with large and uniform surface area can be used as a drug delivery system, and the highly hydrated 3D network within them also provides a good environment for cell growth, which has been widely used in clinical practice. Wang et al. (2019) prepared a collagen hydrogel using tilapia fish skin as a raw material, and confirmed that the collagen hydrogel significantly promoted fibroblast proliferation by cell experiments. It was applied to deep second-degree burn wounds in rats, and the results showed that collagen hydrogel could significantly accelerate the healing of the wounds. Liu et al. (2019) used recombinant human type III collagen hydrogel for the treatment of full-thickness wounds in pigs, and the experimental group recovered well, with a mild inflammatory response, higher number of new capillaries than the negative control group at all time points, and a higher proportion of type III collagen in the new dermal collagen. This study demonstrated that recombinant human type III collagen hydrogel could promote wound healing and revascularization, and was expected to reduce scar formation after wound healing.

#### 3.3.3 Collagen films

Films are highly elastic and flexible structures that act as wound barrier to prevent bacterial penetration while allowing gas exchange (Felgueiras and Amorim, 2017). Collagen films can be used clinically as wound dressings to reinforce and regenerate damaged tissues, and as scaffolds for fibroblasts to promote their attachment and proliferation, provide nutrients to support their growth and improve long-term survival (Chattopadhyay and Raines, 2014). Gao et al. (1992) prepared porcine collagen films for application to burn wounds, and compared with petrolatum gauze, porcine collagen film dressings significantly promoted wound healing, while no adverse effects such as wound infection were observed. La et al. (2001) prepared silver sulfadiazine collagen burn film applied to deep burn wounds in rats, and the results showed that the content of hydroxyproline in the experimental group was significantly higher than that in the control group, while maintaining a lower water content, suggesting that the collagen film can promote the lysis of necrotic tissue from the trauma surface and has the effect of promoting the healing of burn wounds.

### 3.4 Hyaluronic acid

#### 3.4.1 Hyaluronic acid sponge

Hyaluronic acid has a large number of carboxyl and hydroxyl groups and exhibits a high degree of hydrophilicity, which enhances exudate absorption and cell adhesion (Graça et al., 2020), and is mostly used in the preparation of sponges and hydrogels (Table 4). Hyaluronic acid sponge dressings have good absorption properties, induce repair cell infiltration and proliferation, and are used for prolonged wound coverage. Currently, research is focused on combining hyaluronic acid with other polymers or chemically modifying it to generate hyaluronic acid derivatives to improve the physicochemical and biological properties of hyaluronic acid sponges (Graça et al., 2020). In one study (Catanzano et al., 2018) tranexamic acid was incorporated into hyaluronic acid sponge to enhance its hemostatic properties and further promote wound healing. Anisha et al. (2013) prepared a chitosan-hyaluronic acid/nanosilver composite sponge with significant antibacterial activity against *Escherichia coli* and *Staphylococcus aureus*, which can be used to treat diabetic foot ulcers infected with drug-resistant bacteria. Huang et al. (2011) prepared an activated degraded agarose-grafted hyaluronic acid (Ag-g-HA) sponge dressing for skin wound repair in mice. The results showed that the repaired tissues were similar to normal tissues and no infection or exudation of body fluids were observed. It was confirmed that the agarose-modified hyaluronic acid sponge has the function of supporting skin regeneration.

**TABLE 4:** The clinical applications of HA.

Raw material	Biomaterial products	Application	References
Hyaluronic acid	sponge dressings	wound coverage	Graça et al. (2020)
	hyaluronic acid hydrogel	skin wound repair	Lam et al. (2014)
tranexamic acid and hyaluronic acid	Sponge dressings	hemostatic	Catanzano et al. (2018)
chitosan-hyaluronic acid/nanosilver	chitosan-hyaluronic acid/nanosilver composite sponge	diabetic foot ulcers infected	Anisha et al. (2013)
activated degraded agarose-grafted hyaluronic acid (Ag-g-HA)	Sponge dressings	skin wound repair	Huang et al. (2011b)
modified hyaluronic acid, poly-ε-L-lysine and poloxamer F127	injectable hydrogels with self-healing properties	skin wound repair	Wang et al. (2019b)
hyaluronic acid and ZIF-8 nanoparticles	films	skin wound repair	Abednejad et al. (2019)
hyaluronic acid, polycaprolactone, chitosan and zeaxolysin	a bilayer electrostatic spinning film	skin wound repair	Figueira ad Miguel, (2016)

#### 3.4.2 Hyaluronic acid hydrogel

Hyaluronic acid-based hydrogels have good biocompatibility and can provide a moist environment to influence the external microenvironment of the wound, as well as have the ability to promote cellular infiltration and proliferation (Lam et al., 2014), and have a modulating effect on the internal microenvironment of the wound. HA-based hydrogels have been extensively studied for wound dressing applications due to their intrinsic properties, i.e., biocompatibility, ability to provide a moist environment, and promote cell infiltration and proliferation. Wang et al. (2019) combined modified hyaluronic acid with poly-ε-L-lysine and poloxamer F127 to create injectable hydrogels with self-healing properties, loaded with MSC exosomes to promote wound healing and complete re-epithelialization.

#### 3.4.3 Hyaluronic acid films

The presence of three functional groups in the structure of hyaluronic acid, carboxyl, hydroxyl, and acetylamino groups, makes its structure easily modifiable. Current studies on hyaluronic acid films have focused on improving their biological properties by incorporating bioactive molecules and inorganic compounds such as growth factors, natural product extracts, etc. Abednejad et al. (2019) combined ZIF-8 nanoparticles with hyaluronic acid and the mechanical and antimicrobial properties of the films doped with FZIF-8 nanoparticles were enhanced without affecting the adhesion and proliferation of fibroblasts. The preparation of bilayer films by electrostatic spinning technique to mimic the epidermis and dermis of the skin is also a recent research interest (Miguel et al., 2019). The top layer avoids bacterial invasion and wound dehydration, while the bottom layer removes wound exudate and promotes cell infiltration and proliferation (Miguel et al., 2017). Figueira ad Miguel, (2016) fabricated a bilayer electrospun membrane by electrostatic spinning technique, where the top layer consisted of hyaluronic acid and polycaprolactone and the bottom layer consisted of chitosan and zeaxolysin. The membrane exhibited ideal porosity, which, while acting as a barrier to prevent external contamination, facilitated the adhesion, spreading, and proliferation of fibroblasts, which in turn promoted wound healing.

### 3.5 Progress in composite materials research

As shown in the examples above, it has been difficult for a single natural polymer material to meet the requirements of skin healing applications, and most of the existing research has been on composite materials based on natural polymer materials. The main disadvantages of chitosan are its low solubility and poor mechanical properties, which severely limit its clinical application. By compositing with other natural or synthetic materials, it is possible to compensate for its deficiencies while enhancing other properties required for wound healing. Polyvinyl alcohol composites with chitosan have been reported to have improved mechanical properties. Silver ions, which have antimicrobial properties, have been composited with chitosan to enhance the original antimicrobial effect of both and are now used clinically as antimicrobial agents (Azuma et al., 2015; Silva et al., 2015). Chitosan-based composites are important in the field of wound care (Jiménez-Gómez and Cecilia, 2020).

Cellulose has similar problems. In the case of bacterial cellulose, BC is poorly soluble in common solvents due to its high polarity and strong intermolecular hydrogen bonding. At the same time, bacterial cellulose lacks large membrane pores, which is not favorable for cell movement and adhesion, which in turn limits its clinical application. Therefore gelatin, salt, sugar, polyethylene glycol, hydroxyapatite, sodium and calcium ions, are often combined with bacterial cellulose to increase the porosity of biomaterials (Silva et al., 2022). In addition to this the second major drawback related to the medical application of BC is its lack of antimicrobial activity to prevent wound infection. Therefore, several bactericidal elements have been added to BC to enhance its antimicrobial activity (de Oliveira Barud et al., 2016) such as silver (Rai et al., 2009), copper (He et al., 2023), TiO (Gutierrez et al., 2012), propolis (Barud et al., 2013), chitosan (Strnad and Zemljič, 2023).

As a negatively charged natural polysaccharide, HA can interact with positively charged materials to form polyionic complexes that can be used in the form of hydrogels, films and tissue engineering scaffolds (Di Mola et al., 2022). Such as the cationic polysaccharide chitosan (Raik et al., 2020).

Collagen deposition *in vitro* is too slow along with poor mechanical properties (Benny et al., 2016). Collagen/chitosan-PU hybrid scaffolds were selected to mimic the natural ECM with collagen and chitosan, while the addition of TPU improved the mechanical properties of the scaffold material. The study showed that the scaffolds were flexible with high tensile strength and good biocompatibility (Huang C. et al., 2011).

### 3.6 Innovation in drug carriers

#### 3.6.1 Nanocarriers

Nanoparticles (NPs) are the basic components of nanostructures with their unique sizes and properties. The applications of NPs in the field of skin healing are mainly drug delivery, tissue engineering, which can be specifically categorized into metal and metal oxide nanomaterials, non-metallic nanomaterials. Compared with traditional drug delivery systems, nanoparticles as a localized drug delivery system can achieve higher local drug concentration, improved physicochemical stability of the drug loaded in nanoparticles, and sustained and controlled drug delivery from nanoparticulate systems (Ghasemiyeh and Mohammadi-Samani, 2020). This makes drug therapy more effective (Bai et al., 2020). Metallic nanomaterials are mainly gold (Chen and Feng, 2022), silver, copper, zinc oxide, and non-metallic nanomaterials such as aminophenyl sulfone (DAP) (Schneider-Rauber et al., 2020), lipids (Sala et al., 2018). Nanostructured lipid carriers can protect the drug and control the drug release on the one hand, and on the other hand, they can increase the residence time of the localized wound, thus reducing the healing time (Ghasemiyeh and Mohammadi-Samani, 2020).

#### 3.6.2 Smart hydrogels

Hydrogel is a three-dimensional crosslinked polymer network system with water as the dispersion medium (Bao et al., 2019). With high inclusivity, it can be used as a carrier for various bioactive factors. In recent years, various types of smart hydrogels, such as environmentally responsive, self-repairing, self-assembled conductive, shape memory and supramolecular hydrogels, have been rapidly developed to enhance their biomaterial properties and compensate for some of their shortcomings as drug carriers, greatly expanding the application areas of hydrogels. Polymer hydrogels have low mechanical strength and multiple stimuli-triggered responses, and in this context, self-repairing hydrogels have been developed, which can repair themselves when ruptured or traumatized, and have better stability and durability. Qian et al. prepared a self-repairing and injectable hydrogel with a composite material containing chitosan, filipin protein and PRP, which was shown to promote repair cell proliferation *in vitro* and accelerate wound healing in a type II diabetic rat model (Qian et al., 2020).

## 4 Conclusion

Collagen, hyaluronic acid, and chitosan have good biocompatibility and can affect the internal microenvironment of wounds by inducing cellular signals, recruiting neutrophils, enhancing macrophage phagocytosis, and other biological pathways to effectively promote wound healing. Because the internal wound microenvironment cannot be directly and continuously observed, the specific processes of polymer effects on wounds have not been described in detail. Currently fluorescent staining for tracking and monitoring of cells does not meet our needs for observing interactions between cells and biomaterials. For the sake of brevity, only recent advances in the use of some representative naturally derived materials for wound healing have been discussed. More basic research and experimental techniques are needed to support it.

However, natural polymeric materials also have some problems when used as wound healing materials because they are naturally formed and not chemically modified, such as poor mechanical properties of collagen and rapid degradation rate. Hyaluronic acid has poor mechanical properties and water resistance; the mechanical properties of chitosan are poor, and the degradation ability is general, which limits the application of natural polymer materials. In response to this, the current research has two solutions. The first is to combine natural polymer materials with other polymers and make certain chemical modifications to create more multifunctional materials to obtain more suitable properties. The technical difficulty lies in the fact that there is no consensus on the optimal ratio between different polymers, and because the proportion of each component in these mixtures is different, it is impossible to accurately describe the exact effect of each component on the therapeutic effect, which still needs to be supported by a large amount of experimental data.

Another research direction is to reform drug carriers, such as nanoscale engineering scaffolds can strengthen the control of cell space organization, hydrophilic and lipophilic carriers can improve their stability by encapsulating drugs (Huang and Fu, 2010), and design carriers for different application needs to achieve satisfactory clinical results. To date, this is still a huge research gap that requires the joint attention and efforts of researchers from different fields.
